# Haemodynamic and clinical impacts of switching phosphodiesterase-5 inhibitors to riociguat in patients with chronic thromboembolic pulmonary hypertension (CTEPH) after balloon pulmonary angioplasty (BPA) – a prospective cohort study

**DOI:** 10.1186/s12890-025-04069-y

**Published:** 2026-01-03

**Authors:** Timothy Ho Him Kam, Kevin Ka Ho Kam, Michael Ka Lam Wong, Bryan Ping Yen Yan, Guangming Tan

**Affiliations:** 1 Division of Cardiology, Department of Medicine & Therapeutics, Prince of Wales Hospital, Shatin, Hong Kong; 2 Division of Cardiology, Department of Medicine & Therapeutics, Faculty of Medicine, The Chinese University of Hong Kong, Shatin, Hong Kong; 3 Cardiac Medical Unit, Grantham Hospital, Aberdeen, Hong Kong

**Keywords:** Chronic thromboembolic pulmonary hypertension, Balloon pulmonary angioplasty, Riociguat

## Abstract

**Background:**

For patients with chronic thromboembolic pulmonary hypertension (CTEPH), balloon pulmonary angioplasty (BPA) has been associated with superior reductions in mean pulmonary artery pressure (mPAP) and pulmonary vascular resistance (PVR) when compared to riociguat. In patients with pulmonary arterial hypertension (PAH), greater clinical improvements were observed after switching from phosphodiesterase-5 inhibitors (PDE5i) to riociguat. However, the impact of transitioning from PDE5i to riociguat on pulmonary haemodynamics and functional outcomes after BPA remains unclear.

**Methods:**

This prospective, open-label, single-arm, study enrolled CTEPH patients who remained symptomatic following BPA. After a 24-hour PDE5i washout period, patients were switched to riociguat. At week 26, primary outcomes assessed changes in haemodynamics including PVR and mPAP. Secondary endpoints evaluated cardiac index; functional status including WHO functional class, 6-minute walking distance (6MWD), REVEAL Lite 2 score; biochemical markers such as N-terminal prohormone of brain natriuretic peptide (NT-proBNP); and echocardiographic measurements of right-heart function. Treatment-related adverse events and clinical worsening were monitored throughout the study.

**Results:**

From July 2024 to January 2025, 16 patients (mean age 62.3 ± 14.6 years; 75% female) were recruited, with 14 completing the 26-week follow-up. At week 26, significant reductions occurred in PVR (-2.16 Wood units; CI -3.64 to -0.69; *p* = 0.007) and mPAP (-4.79 mmHg; Confidence Interval [CI] -8.05 to -1.52; *p* = 0.007). Significant improvements were also noted in cardiac index, WHO functional class, 6MWD, REVEAL Lite 2 score and NT-proBNP levels. Echocardiographic measurements of right-heart function did not demonstrate significant improvement. Treatment-related adverse events were observed in 11 patients (68.75%). Clinical worsening occurred in four patients, including two deaths unrelated to treatment and two unplanned hospitalisations due to pulmonary hypertension.

**Conclusion:**

In CTEPH patients after completion of BPA, replacing PDE5i with riociguat significantly enhanced pulmonary haemodynamics and functional capacity but was accompanied by a considerable risk of treatment-related adverse events.

**Trial registration:**

ClinicalTrials.gov Identifier NCT06715280 retrospectively registered on 26/11/2024.

## Introduction

 Surgical pulmonary endarterectomy (PEA) is the preferred treatment for chronic thromboembolic pulmonary hypertension (CTEPH) patients with accessible pulmonary artery occlusions [1, 2]. For those with inoperable CTEPH or persistent pulmonary hypertension after PEA, current treatment options include balloon pulmonary angioplasty (BPA) and medical therapies. Several medical therapies that target the microvascular aspects of pulmonary arterial hypertension (PAH), such as phosphodiesterase-5 inhibitors (PDE5is) and endothelin receptor antagonists (ERAs), have been used off-label in patients with inoperable CTEPH, as their effectiveness has not been proven through randomised controlled trials in this patient cohort [3]. Riociguat, a soluble guanylate cyclase stimulator (sGCs), is the first agent approved for treating symptomatic patients with inoperable CTEPH based on the results from the CHEST-1 trial, which showed that riociguat significantly reduced pulmonary vascular resistance and improved exercise capacity in patients with inoperable CTEPH or persistent/recurrent pulmonary hypertension after PEA [4].

Two clinical trials have demonstrated improvements in clinical parameters and biochemical markers after switching PDE5i to sGCs in selected patients with PAH who had an inadequate response to PDE5i [5, 6]. While the application of this switching strategy in patients with CTEPH has not been validated in large clinical trials, a smaller prospective study [7] demonstrated the safety and effectiveness in reducing brain natriuretic peptide (BNP) levels with this approach.

In addition to medical treatments, balloon pulmonary angioplasty (BPA) has emerged as a treatment option for patients with inoperable CTEPH or for those experiencing persistent or recurrent pulmonary hypertension after PEA [8]. Two randomised controlled trials comparing BPA with riociguat have demonstrated that BPA was associated with a greater reduction in mean pulmonary artery pressure (mPAP) and pulmonary vascular resistance (PVR) in inoperable CTEPH patients [9, 10]. However, it remains unclear whether switching from PDE5i to riociguat provides additional functional and haemodynamic benefits after the completion of BPA. Therefore, this study was designed to evaluate the safety and efficacy of switching from PDE5i to riociguat in patients with CTEPH who have completed BPA treatment.

## Methods

### Study Design

This study was an investigator-initiated, prospective, open-label and single-centre cohort study. It was carried out in accordance with the principles of the Declaration of Helsinki, and written informed consent was obtained from all participating patients.

### Patient selection

Patients with CTEPH, as defined by the ESC guideline [3], were recruited into this study if they fulfilled all of the following criteria: (1) Remained symptomatic with WHO functional class II or III after completion of balloon pulmonary angioplasty (BPA); (2) On stable and maximally tolerated dose of sildenafil for at least 6 weeks as monotherapy, or in combination with other pulmonary hypertension specific therapies; (3) No escalation in diuretic dosage within 30 days; and (4) No recent hospitalisation due to pulmonary hypertension or heart failure within 30 days.

Patients were excluded from this study if they met any of the following criteria: (1) Use of nitrates or nitric oxide donors via any route of administration within 30 days; (2) Pregnant or breast-feeding women; (3) Renal impairment with glomerular filtration rate < 15mL/min/1.73 m^2^; (4) Child-Pugh C hepatic impairment; and (5) Systolic blood pressure below 95mmHg.

### Study protocol

**Fig. 1:**
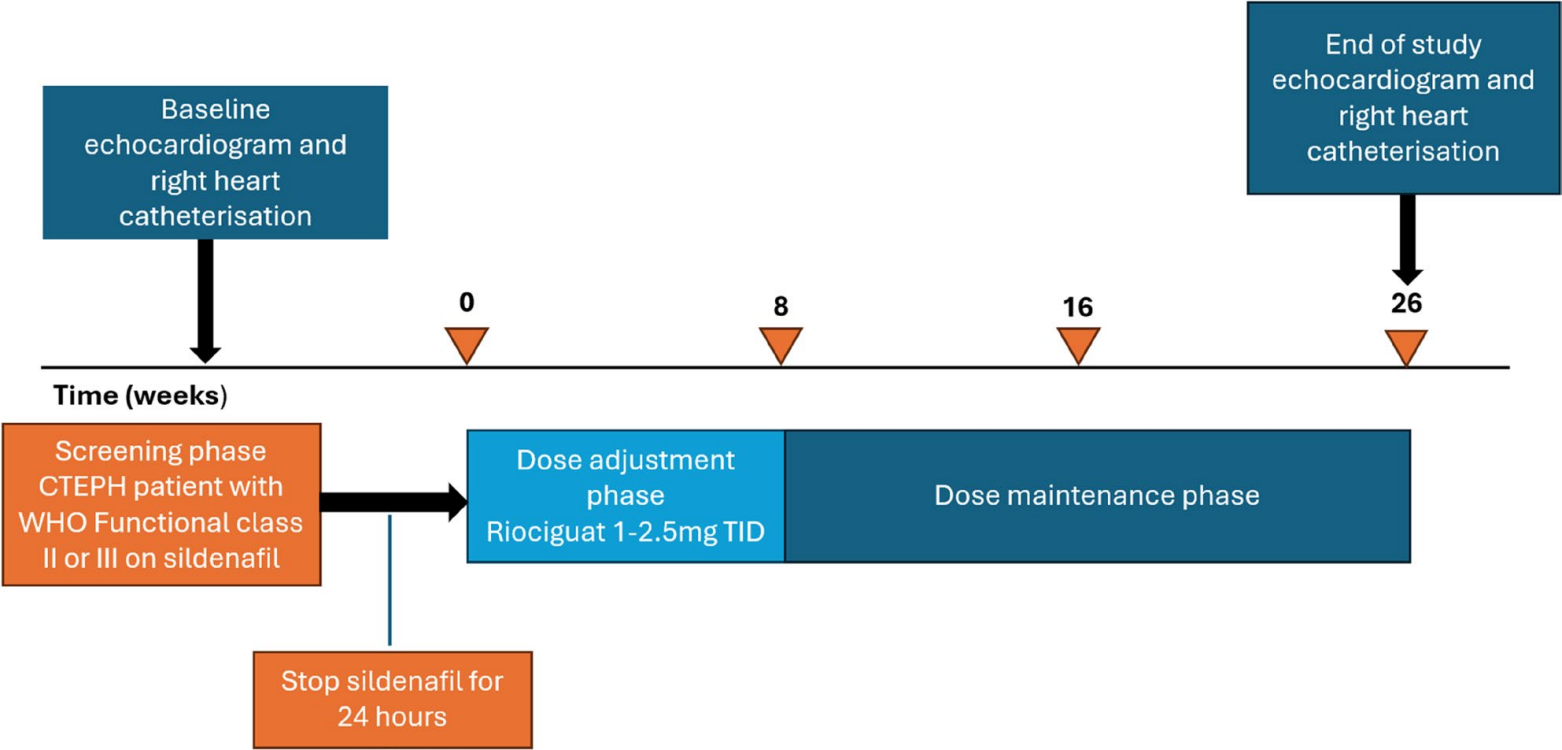
Study workflow

This study consists of baseline evaluations, a 24-hour washout period for PDE5i, and a 26-week follow-up which consisted of an 8-week period for riociguat titration, and an 18-week maintenance phase. The overall study workflow is outlined in Fig. 1.

Riociguat (Bayer AG, Kaiser-Wilhelm-Allee, Leverkusen, Germany) was administered orally using a dose titration regimen in accordance with the United States Food and Drug Administration (FDA) prescribing guidelines: After a 24-hour washout from sildenafil, riociguat was initiated at 1 mg three times daily (TID), with incremental increases of 0.5 mg every two weeks (up to a maximum of 2.5 mg TID) if systolic blood pressure remained at or above 95 mmHg. Dose escalation occurred over an 8-week period. The dose was reduced by 0.5 mg TID if there were symptoms of hypotension. Concomitant use of other pulmonary hypertension-specific therapies such as ERAs at baseline was permitted.

Study visits were arranged at baseline, then at weeks 2, 4, 6, 8, 16 and 26. Baseline measurements, including right heart catheterisation, echocardiogram, 6MWD, WHO functional class and NT-proBNP levels, were evaluated for each patient while on sildenafil. WHO functional class and NT-proBNP were assessed at every clinic visit. The 6MWD, along with the Registry to Evaluate Early and Long-Term PAH Disease Management (REVEAL^®^) Lite 2 score, were evaluated at baseline, weeks 8, 16, and 26. Right heart catheterisation and echocardiogram were repeated at week 26. Safety and treatment-related adverse events were monitored throughout the study. All patients received standard medical care according to the latest treatment guidelines, and treatment escalations were permitted at the clinician’s discretion.

### Outcome measures

The primary endpoints of this study were the changes in PVR and mPAP from baseline to week 26. The secondary endpoints included the changes in cardiac index, WHO functional class, NT-proBNP levels, 6-minute walking distance (6MWD), REVEAL Lite 2 risk score, tricuspid regurgitation pressure gradient (TRPG) and tricuspid annular plane systolic excursion (TAPSE).

Safety outcomes including treatment-related adverse events and clinical worsening were assessed throughout the study. Clinical worsening was defined as all-cause mortality, heart or lung transplantation, salvage PEA or BPA due to deterioration of the primary disease, unplanned hospitalisations related to pulmonary hypertension (PH), initiation of new PH treatments, a sustained decline of more than 15% from baseline or over 30% from the most recent measurement in 6MWD, persistent worsening of WHO functional class due to deterioration of the primary disease, symptoms or signs of right heart failure not responding to optimised oral diuretic therapy, with reference to the criteria of clinical worsening established in the CHEST-1 study of riociguat [4].

#### Statistical analysis

All data for continuous variables were expressed as mean values ± standard deviation, and categorical variables were expressed as numbers with percentages. The Shapiro-Wilk test was used to assess the normality of continuous variables, including the PVR, mPAP, cardiac index, 6MWD, NT-proBNP, TAPSE and TRPG. Changes from baseline to week 26 for these variables were then analysed using a paired samples t-test for normally distributed data, or the Wilcoxon signed-rank test for non-normally distributed data. The Wilcoxon signed-rank test was also employed to evaluate the statistical significance of changes in the WHO functional class and REVEAL Lite 2 score. For any patient who died or withdrew due to clinical worsening, the last available measurements, including the 6MWD, NTproBNP, WHO functional class and REVEAL Lite 2 score, were carried forward for all analyses where feasible. A hierarchical testing procedure was adopted to address multiplicity in endpoints analysis. The endpoints were tested in the following order: (1) PVR, (2) mPAP, followed by (3) WHO functional class, (4) 6MWD, (5) NT-proBNP, (6) cardiac index, (7) REVEAL Lite 2 score, (8) TAPSE and (9) TRPG. Formal hypothesis testing for a given endpoint was only performed if all preceding endpoints in the sequence were statistically significant, defined as two-sided p-values less than 0.05.

## Results

### Baseline patient characteristics

Between July 2024 and January 2025, 19 patients who completed BPA at our centre since Jan 2019 were screened, and 16 of them (mean age 62.3 ± 14.6 years; 75% female) were recruited. Three patients were excluded: two declined to participate, and one was asymptomatic. Baseline patient characteristics are summarised in Table 1. At baseline, all patients were in WHO functional class III, and 11 patients (68.8%) required long-term oxygen therapy. The mean duration of prior sildenafil therapy was 33.6 months. Among 16 patients, 15 (93.8%) were on concomitant endothelin receptor antagonists (14 [87.5%] macitentan and 1 [6.3%] bosentan). Two patients were on triple therapy, with the addition of prostacyclin analogue or prostacyclin receptor agonist.

Between January 2019 and April 2024, our centre performed 37 BPA procedures. Of the 16 patients enrolled, the average number of BPA sessions per patient was 2.1. Treatment was concluded for 10 patients who had no further treatable lesions, while three others had only challenging chronic total occlusions remaining. The remaining three patients declined further intervention. The mean interval between the last BPA session and study enrolment was 10.8 months.

**Table 1:** Patient baseline characteristics

Baseline Characteristics	*N* = 16
Age, years	62.3 ± 14.6
Sex	
Male	4 [25%]
Female	12 [75%]
BMI (Kg/m^2^)	23.5 ± 4.2
SBP (mmHg)	113.6 ± 13.2
Heart rate (beats per minute)	82.9 ± 16.8
Long-term oxygen therapy	11 [68.8%]
Treatment duration with sildenafil (months)	33.6 ± 22.7
Concomitant therapies	
ERA	15 [93.8%]
Prostacyclin receptor agonist	1 [6.3%]
Prostacyclin analogue	1 [6.3%]
Diuretics	8 [50%]
Medical Comorbidities	
Hypertension	2 [12.5%]
Diabetes mellitus	4 [25%]
Ischaemic stroke	1 [6.3%]
Intracranial haemorrhage	1 [6.3%]
Ischaemic heart disease	1 [6.3%]
Malignancy	0 [0%]
Chronic kidney disease	4 [25%]
Autoimmune disease	1 [6.3%]
Systemic lupus erythematosus	1 [6.3%]
Interstitial lung disease	3 [18.75%]
BPA procedural characteristics	
Number of BPA procedures	2.1 ± 1.5
Number of segmental pulmonary arteries undergone BPA	4.4 ± 3.1
Final BPA till enrolment (months)	10.8 ± 4.7
WHO functional class	
II	0 [0%]
III	16 [100%]
6MWD (m)	236.9 ± 130.5
NT-proBNP (ng/L)	1370.2 ± 1853.0
REVEAL Lite 2 score (Out of 14)	7.3 ± 2.3
Echocardiographic parameters	
TRPG (mmHg)	61.7 ± 24
TAPSE (mm)	17.7 ± 3.8
Haemodynamics variables	
mPAP (mmHg)	45.2 ± 11.3
PVR (Wood units)	11.1 ± 5.8
CO (L/minute)	3.5 ± 1.1
Cardiac Index (L/minute/m^2^)	2.2 ± 0.7
PAWP (mmHg)	11.4 ± 5.1

Data are presented as n [%] or mean ± SD

*BMI* Body mass index, *SBP* Systolic blood pressure, *ERA* Endothelin receptor antagonist, *WHO* World Health Organisation, *PAH* Pulmonary arterial hypertension, *PH* Pulmonary hypertension, *BPA* Balloon pulmonary angioplasty, *REVEAL* The Registry to Evaluate Early and Long-Term PAH Disease Management, *TRPG* Tricuspid regurgitation pressure gradient, *TAPSE* Tricuspid annular plane systolic excursion, *6MWD* 6-min walking distance, *NT-proBNP* N-terminal prohormone of brain natriuretic peptide, *mPAP* mean pulmonary arterial pressure, *PVR* Pulmonary vascular resistance, *CO* Cardiac output, *PAWP* Pulmonary artery wedge pressure

**Fig. 2:**
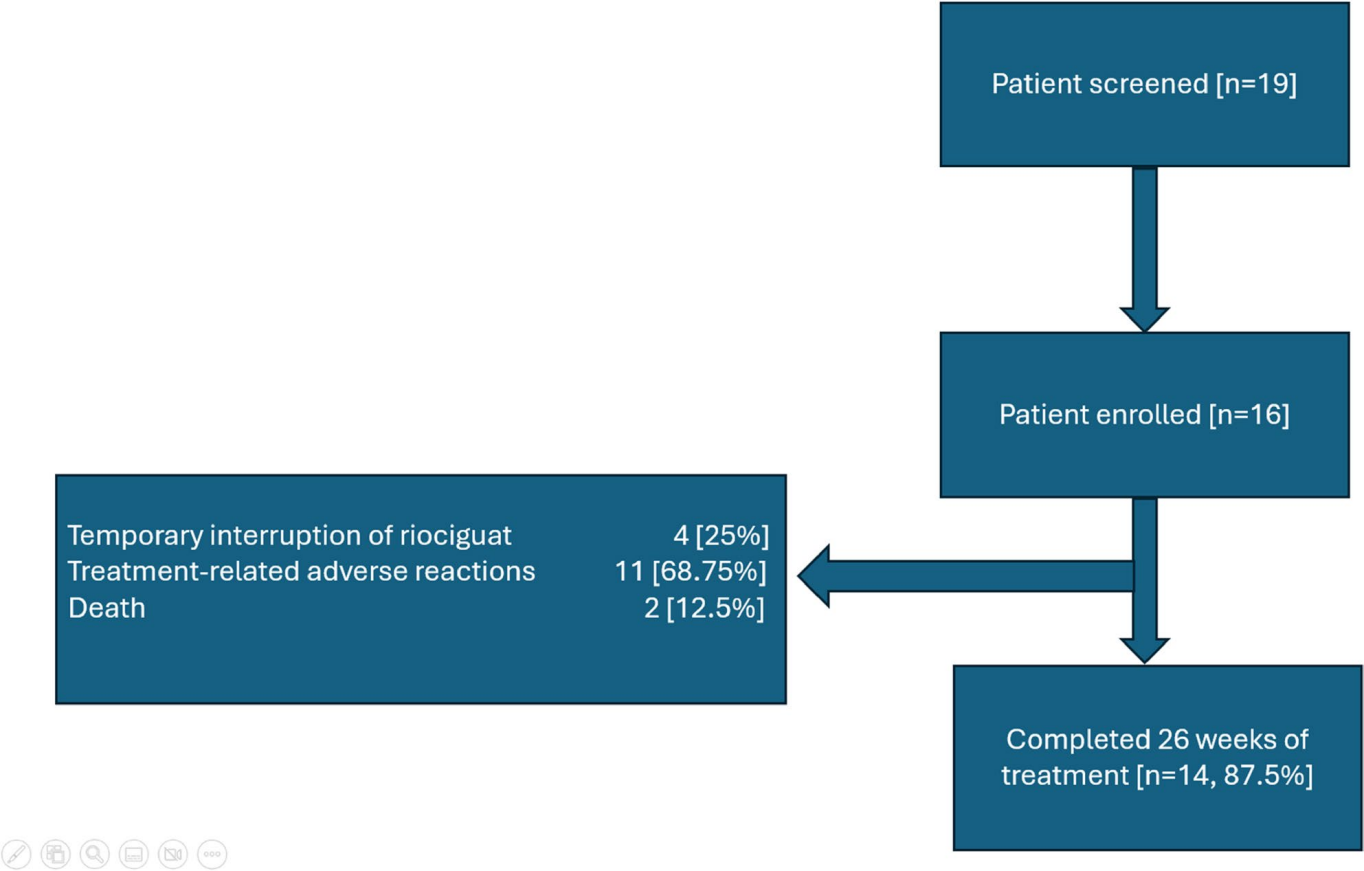
Trial profile. Among 16 patients enrolled, 14 (87.5%) completed the 26-week follow-up

**Fig. 3:**
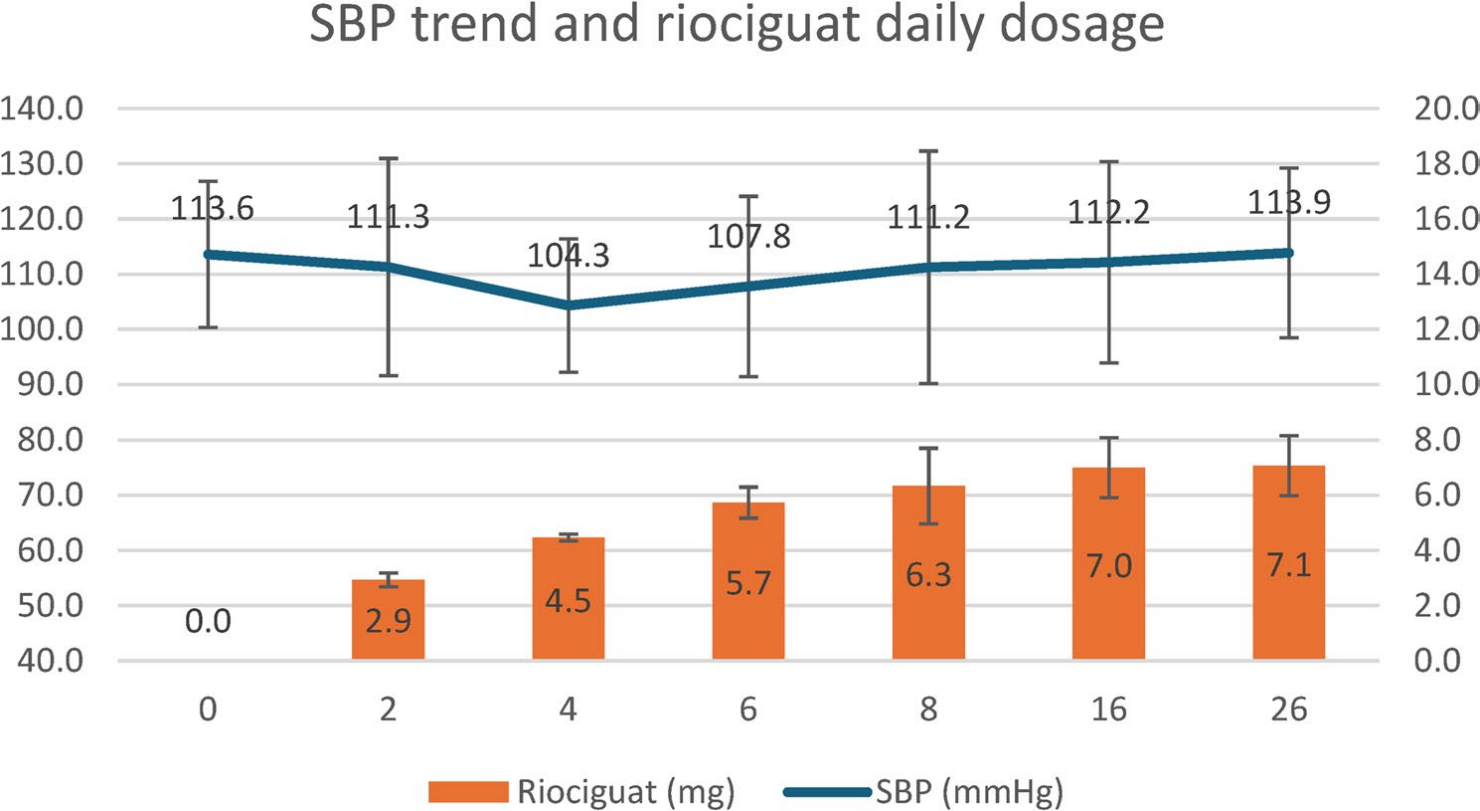
The systolic blood pressure (SBP) trend and the dosage of riociguat per day during the 26-week study

Of the 16 patients enrolled in this study, 14 (87.5%) completed the 26-week follow-up. The trial profile is shown in Fig. 2. The two discontinuations were due to mortality. At week 26, 12 patients (86%) reached the maximum dose of riociguat (2.5 mg TID), while the remaining two (14%) were maintained at 1.5 mg TID due to systemic hypotension. Figure 3 illustrates the systolic blood pressure (SBP) trend during the study. A nadir in mean SBP (104 mmHg) was observed at week 4 when the dose of riociguat was increased to 4.5 mg per day. After that, the mean SBP appeared to be stabilised with further titration of riociguat.

### Primary endpoints

**Fig. 4:**
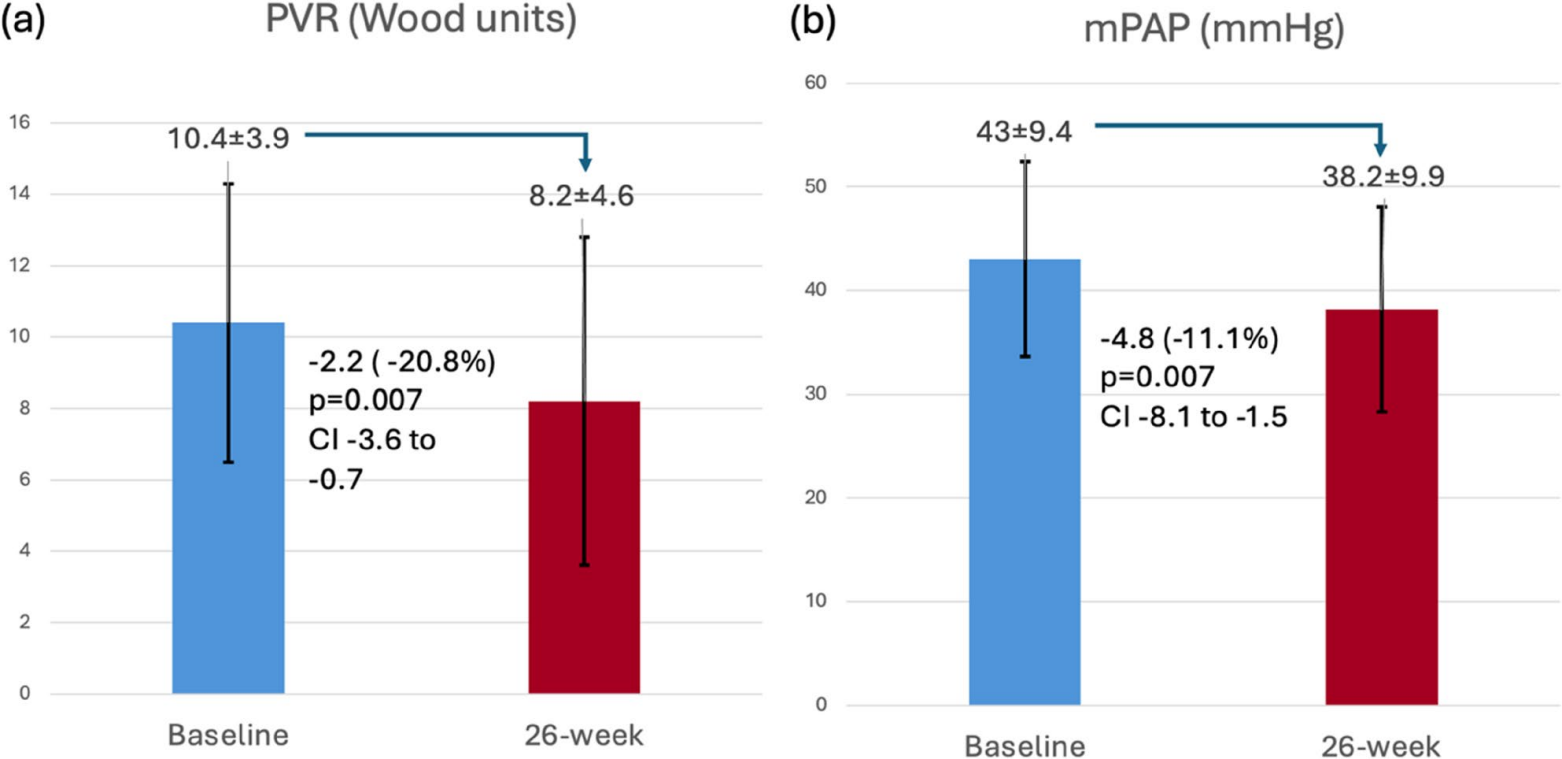
Effect of riociguat on primary endpoints: (**a**) PVR and (**b**) mPAP. Data are presented as mean ± SD

Significant improvements were observed in the primary endpoints among the 14 patients who completed 26 weeks of treatment (Fig. 4 and Table 2). The mean PVR decreased by 20.8% (−2.16 ± 2.55 Wood units; 95% CI −3.64 to −0.69; *p* = 0.007) and the mPAP decreased from baseline by 11.1% (absolute reduction − 4.79 ± 5.66 mmHg; 95% Confidence Interval, CI, −8.05 to −1.52; *p* = 0.007).

**Table 2:** Analysis of change in primary endpoints

Parameter	*N*	Value at baseline	Value at 26-week	Change from baseline	*p*-value for mean change from baseline (95% CI)
PVR, Wood units	14	Mean 10.39 ± 3.86 Median 9.2 (6.93–13.82) Range 4.07–16.1	Mean 8.23 ± 4.62 Median 7.14 (4.53–10.33) Range 3.23–18.3	Mean − 2.16 ± 2.55 Median − 2.66 (−4.33 -−0.97) Range − 5.24 - +3.0	*p* = 0.007 (−3.64 to −0.69)
mPAP, mmHg	14	Mean 43 ± 9.42 Median 41.5 (34.75–50) Range 31–63	Mean 38.21 ± 9.85 Median 39.5 (30–46.5) Range 21–53	Mean − 4.79 ± 5.66 Median − 4.0 (−10.25 - −2.25) Range − 14 - +6	*p* = 0.007 (−8.05 to −1.52)

Data are presented as mean ± SD or median (interquartile range). *mPAP* mean pulmonary arterial pressure;, *PVR* Pulmonary vascular resistance

### Secondary endpoints

**Fig. 5:**
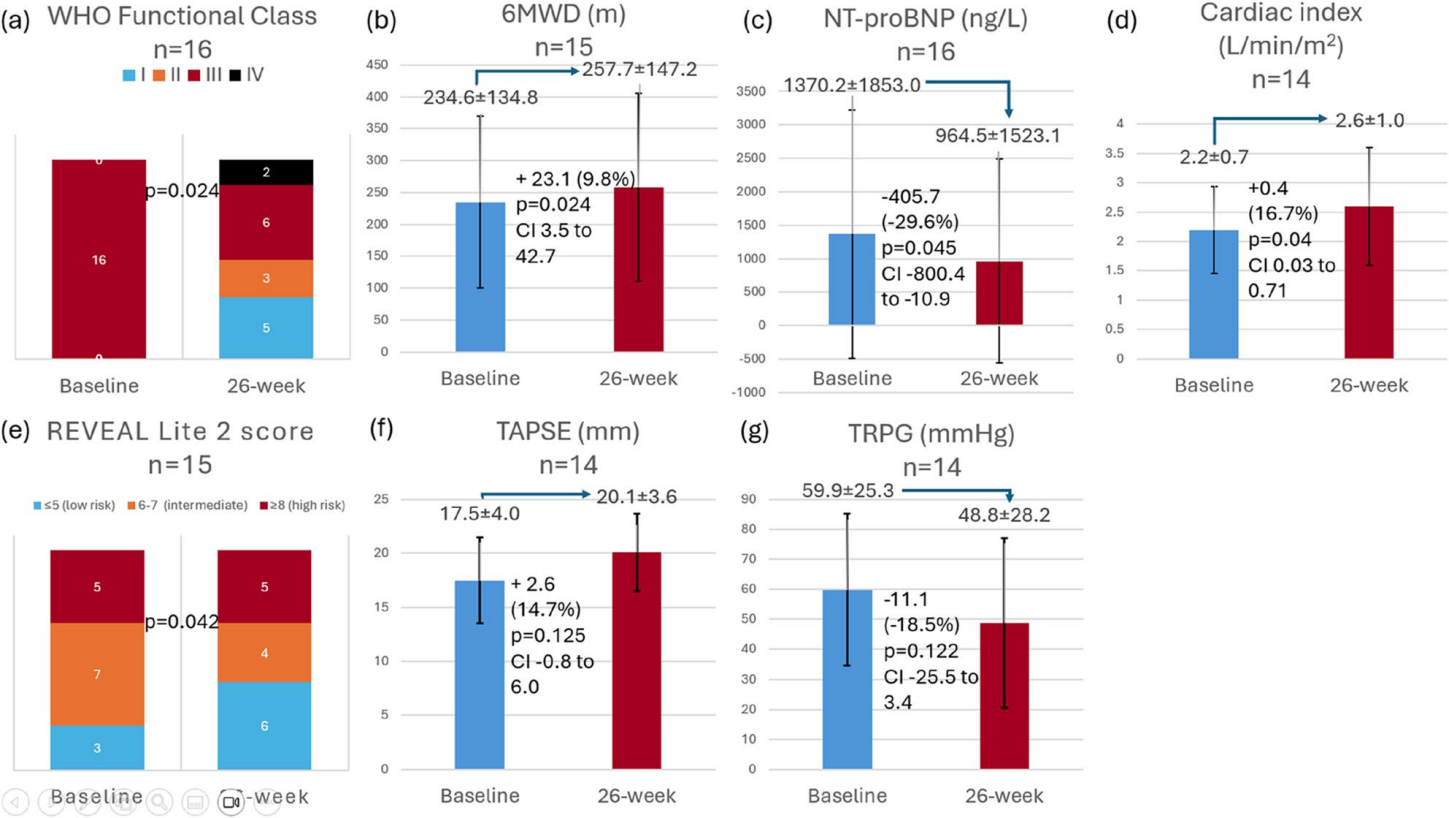
Effect of riociguat on secondary endpoints: (**a**) WHO Functional class; (**b**) 6MWD; (**c**) NT-proBNP; (**d**) Cardiac index; (**e**) REVEAL Lite 2 score; (**f**) TAPSE and (**g**) TRPG. Data are presented as mean ± SD

Changes in secondary endpoints from baseline to week 26 are summarised in Fig. 5 and Table 3. The WHO functional class showed significant improvement, with 5 patients (31.3%) achieving class I and 3 (18.8%) improving to class II. However, 6 patients (37.5%) remained in class III, while 2 (12.5%) deteriorated to class IV. The mean 6MWD significantly improved by 23.09 ± 35.35 m (95% CI 3.51 to 42.66; *p* = 0.024). NT-proBNP levels significantly reduced by 29.6% (−405.69 ± 740.80 ng/L; 95% CI − 800.43 to −10.94; *p* = 0.045). The cardiac index increased significantly by 16.7% (0.37 ± 0.59 L/min/m^2^; 95% CI 0.03 to 0.71; *p* = 0.036). The mean REVEAL Lite 2 score (out of a total score of 14) decreased by 1.00 ± 1.69 (*p* = 0.042) Tables 2 and 3.

The echocardiographic parameters also demonstrated improvement, but the changes did not reach statistical significance. The TAPSE increased by 14.7% (2.58 ± 5.89 mm; 95% CI −0.82 to + 5.98; *p* = 0.125), while the TRPG decreased by 18.5% (11.07 ± 25.04 mmHg; 95% CI −25.52 to + 3.39; *p* = 0.122).

**Table 3:** Analysis of change in secondary endpoints

Parameter	*N*	Value at baseline	Value at 26-week	Change from baseline	*p*-value for mean change from baseline (95% CI)
WHO functional class	16	Mean 3.0 ± 0 Median 3.0 (3.0–3.0) Range 3.0–3.0	Mean 2.31 ± 1.08 Median 2.5 (1.0–3.0) Range 1.0–3.0	Mean − 0.69 ± 1.08 Median − 0.5 (−2.0–0.0) Improved/Stable/Worsened 8/6/2	*p* = 0.024
6WMD, m	15	Mean 234.61 ± 134.75 Median 222.6 (129.0–360.0) Range 30–442	Mean 257.69 ± 147.17 Median 299.0 (136.0–353.4) Range 30–511	Mean + 23.09 ± 35.35 Median + 27.0 (0.0–43.0) Range − 64.0 – +76.4	*p* = 0.024 (3.51 to 42.66)
NT-proBNP, ng/L	16	Mean 1370.19 ± 1853.01 Median 639.0 (338–1207.75) Range 31–5749	Mean 964.50 ± 1523.07 Median 306.0 (91.25–1050.25) Range 41–5800	Mean − 405.69 ± 740.80 Median − 227.5 (−504.75 – −4.0) Range − 2953–156	*p* = 0.045 (−800.43 to −10.94)
Cardiac Index, L/minute/m^2^	14	Mean 2.22 ± 0.74 Median 2.37 (1.46–2.80) Range 1.10–3.41	Mean 2.59 ± 1.00 Median 2.73 (1.49–3.24) Range 1.21–4.24	Mean + 0.37 ± 0.59 Median + 0.22 (−0.01 – +0.68) Range − 0.49 – +1.78	*p* = 0.036 (0.03 to 0.71)
REVEAL Lite 2 score (out of 14)	15	Mean 7.27 ± 2.37 Median 7 (6–9) Range 3–11	Mean 6.27 ± 3.20 Median 7 (3–9) Range 2–12	Mean − 1.00 ± 1.69 Median − 1 (−2–0) Improved/Stable/Worsened 8/4/3	*p* = 0.042
TAPSE, mm	14	Mean 17.53 ± 3.97 Median 18.0 (15.38–21.00) Range 9.0–24.0	Mean 20.11 ± 3.60 Median 20.0 (18.75–21.5) Range 13.0–28.0	Mean + 2.58 ± 5.89 Median + 2.0 (−2.13–5.25) Range − 3 – +19	*p* = 0.125 (−0.82 to + 5.98)
TRPG, mmHg	14	Mean 59.89 ± 25.27 Median 53.8 (42.80–72.48) Range 28.00–124.00	Mean 48.82 ± 28.18 Median 51.65 (32.78–71.43) Range 0.67–88.80	Mean − 11.07 ± 25.04 Median 8.00 (−35.6 – +6.15) Range − 49.10 – +32.30	*p* = 0.122 (−25.52 to + 3.39)

Data are presented as mean ± SD or median (interquartile range). *6MWD* 6-minute walking distance, *CI* Confidence interval, *NT-proBNP* N-terminal prohormone of brain natriuretic peptide, *REVEAL* The Registry to Evaluate Early and Long-Term PAH Disease Management, *TAPSE* Tricuspid annular plane systolic excursion, *TRPG* Tricuspid regurgitation pressure gradient, *WHO* World Health Organisation

### Clinical worsening

Four patients experienced predefined clinical worsening events, including two deaths which were not treatment-related: one from pneumonia on day 53 and the other from subdural haematoma following a fall on day 159. Two patients experienced unplanned hospitalisations due to pulmonary hypertension. The first patient was admitted on day 105 due to a loss of consciousness, and the second patient was admitted on day 119 due to right heart failure. No patients required salvage PEA or BPA due to disease progression, nor did they need initiation of new PH therapies or escalation of existing treatments.

### Treatment-related adverse events

11 patients (68.75%) reported at least one treatment-related adverse drug event during the study. The most common adverse events included hypotension (*n* = 4; 25%), nasopharyngitis (*n* = 3; 18.75%), dyspepsia (*n* = 2; 12.5%) and vomiting (*n* = 2; 12.5%). The details of the adverse events are provided in Table 4. Four adverse events resulted in temporary interruption of riociguat, but no permanent discontinuations occurred during the study.

**Table 4:** Treatment-related adverse events

	*N* = 16
Treatment-related adverse events	
Total number of patients with 1 or more adverse events	11 (68.75%)
Hypotension	4 (25%)
Nasopharyngitis	3 (18.75%)
Dyspepsia	2 (12.5%)
Vomiting	2 (12.5%)
Diarrhoea	1 (6.25%)
Dizziness	1 (6.25%)
Headache	1 (6.25%)
Palpitations	1 (6.25%)
Right ventricular failure	1 (6.25%)
Peripheral oedema	0 (0%)
Adverse events leading to the interruption of riociguat	
Any	4 (25%)
Dyspepsia	1 (6.25%)
Headache	1 (6.25%)
Nasopharyngitis	1 (6.25%)
Vomiting	1 (6.25%)

Data are presented as n [%]

### Risk-benefit analysis

Table 5 presents the haemodynamic and functional outcomes for the 11 patients who experienced at least one adverse drug event (ADE). This subgroup achieved reductions in PVR and mPAP of 19.6% and 11.2%, respectively, alongside a 16.6 m improvement in 6MWD and a 37.3% decrease in NT-proBNP. However, the occurrence of ADEs, such as hypotension and nasopharyngitis, necessitated clinical management, including temporary interruption of riociguat or the use of symptomatic medications.

**Table 5:** Risk-benefit analysis

Parameter	*N*	Mean value at baseline	Mean value at 26-week	Change from baseline
PVR, Wood units	10	11.00 ± 3.31	8.84 ± 4.84	−2.16 ± 2.94 (−19.6%)
mPAP, mmHg	10	45.4 ± 9.88	40.3 ± 10.67	−5.10 ± 6.72 (−11.2%)
6WMD, m	11	221.35 ± 119.34	237.95 ± 120.00	+ 16.60 ± 35.67 (+ 7.55%)
NT-proBNP, ng/L	11	1334.82 ± 1680.65	836.91 ± 910.21	−497.91 ± 879.03 (−37.3%)

The evaluation of haemodynamic and functional improvements in patients who experienced at least one adverse drug event. Data are presented as mean ± SD. *6MWD* 6-minute walking distance, *CI* Confidence interval, *mPAP* mean pulmonary arterial pressure, *NT-proBNP* N-terminal prohormone of brain natriuretic peptide, *PVR* Pulmonary vascular resistance

## Discussion

This was the first study to evaluate the safety and efficacy of switching PDE5i to riociguat in patients with CTEPH following BPA. The transition from PDE5i to riociguat resulted in significant improvements in pulmonary haemodynamics, including reductions in PVR and mPAP, as well as favourable changes in cardiac biomarkers and functional capacities, such as WHO functional class and 6MWD. These findings are consistent with the results of the landmark CHEST-1 trial, which demonstrated that riociguat significantly improved PVR, NT-proBNP levels, 6MWD and WHO functional class in inoperable CTEPH patients [4]. Specifically, the CHEST-1 trial reported reductions in PVR (226 ± 248 dyn·s·cm^− 5^ or 2.83 ± 3.1 Wood units) and mPAP (4 ± 7 mmHg), comparable to the reductions observed in our study.

**Fig. 6:**
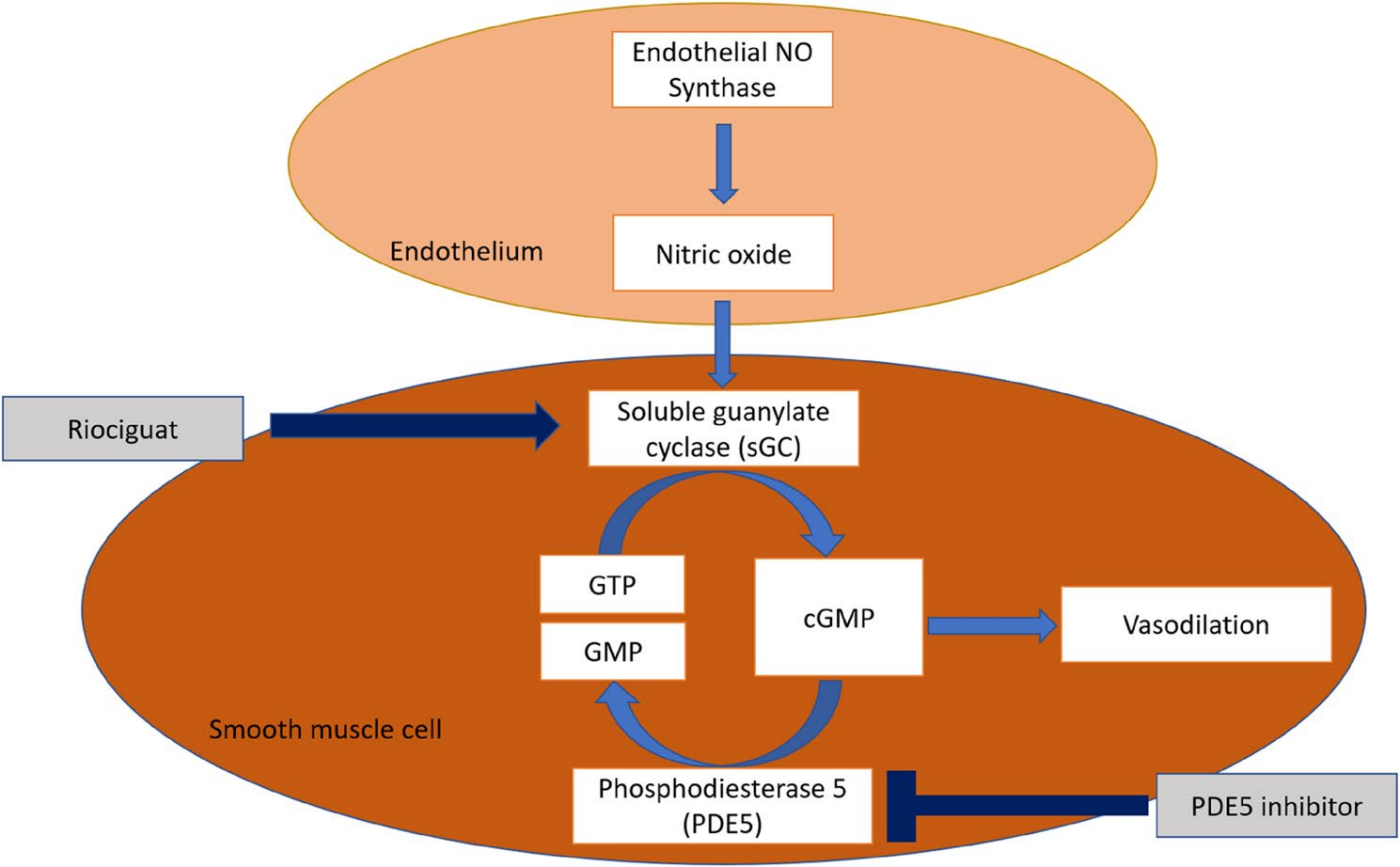
Mechanism of actions of riociguat and PDE5 inhibitor. cGMP: cyclic guanosine monophosphate; GMP: guanosine monophosphate; GTP: guanosine triphosphate; NO: nitric oxide; sGC: soluble guanylate cyclase; PDE: phosphodiesterase

Pulmonary hypertension is associated with endothelial dysfunction of the pulmonary vasculature and reduced expression of endothelial nitric oxide synthase, leading to diminished activation of the soluble guanylate cyclase (sGC) pathway and subsequent pulmonary vasoconstriction [11]. The pathophysiology of CTEPH involves obstructive fibrotic thrombi in the pulmonary arteries [12], along with microvasculopathy, including plexiform lesions, a feature also seen in PAH [13]. Given this shared pathology, medical therapies that are approved for PAH, such as PDE5i, have been used off-label in CTEPH. Nevertheless, this off-label treatment in inoperable CTEPH only holds a Class IIb recommendation from the current ESC guideline [3], because clinical evidence supporting their efficacy in CTEPH remains limited. The existing evidence for the use of sildenafil includes a small randomised controlled trial involving 19 CTEPH patients, which showed that sildenafil improved PVR and WHO functional class but not exercise capacity [14]. In contrast, riociguat has shown significant reductions in PVR and improvements in exercise capacity in both PAH (PATENT-1 trial) and inoperable CTEPH (CHEST-1 trial) [4, 15]. While both PDE5i and riociguat operate through the same nitric oxide (NO)-soluble guanylate cyclase (sGC)-cyclic guanosine monophosphate (cGMP) pathway to promote pulmonary vasodilation, they target different molecular mechanisms. PDE5i works by inhibiting the breakdown of cGMP, thereby prolonging the actions of cGMP to promote smooth muscle relaxation [16]. In contrast, riociguat offers a dual mechanism of action that includes direct stimulation of sGC and sensitisation of sGC to endogenous NO, resulting in smooth muscle relaxation and pulmonary vasodilation [17]. The mechanisms of action of both PDE5i and riociguat are summarised in Fig. 6. Given this pharmacological advantage, replacing PDE5i with riociguat has emerged as a potential therapeutic strategy in PAH and CTEPH. In the present study, patients who had sildenafil for a mean duration of 33.6 months and completed BPA showed significant improvements in haemodynamics and exercise capacity after switching to riociguat. These findings strengthen the evidence that transitioning from PDE5i to riociguat provides clinical benefits for CTEPH patients.

Despite demonstrating efficacy in improving haemodynamic and functional parameters, riociguat treatment was associated with a considerable adverse drug events (ADEs) burden in our study population. In this study, 11 patients (68.75%) experienced at least one ADE, aligning with the known safety profile of riociguat (69–92% adverse event incidence) established in the CHEST-1, MR BPA and RACE trials [4, 9, 10]. While headache (15–25%) and dizziness (17–23%) were most prevalent in these landmark studies, our cohort exhibited a different adverse event pattern characterised by higher rates of hypotension (25%) and nasopharyngitis (18.75%). This discrepancy likely reflects differences in patient characteristics, particularly the fact that 93.8% of our patients received concomitant pulmonary vasodilator therapy (ERAs or prostacyclin analogues), and this group of patients were excluded from the landmark trials. A high proportion of our patients were on combination therapies approved for PAH because patients were usually referred late for BPA, and remained symptomatic despite initial treatments including anticoagulation, diuretics and PDE5i. As mentioned earlier, these drug classes approved for PAH were used off-label in CTEPH given the shared microvascular pathology between PAH and CTEPH [3]. Although such combination therapy is often excluded in clinical trials, its inclusion here reflects a cohort not uncommonly encountered in routine clinical practice and illustrates the real-world challenges in this population.

Despite requiring temporary drug interruption or symptomatic management for ADEs, the risk-benefit analysis showed that the affected subgroup achieved meaningful haemodynamic and clinical improvements overall. As a strategy to mitigate hypotensive risk, we propose withholding concomitant pulmonary vasodilators in patients who experience symptomatic hypotension during riociguat titration. This ensures that riociguat, the first-line treatment for inoperable CTEPH, can be maintained. We also suggest frequent home blood pressure monitoring, with closer surveillance for patients who experience hypotension during riociguat titration.

In this study, the incidence of clinical worsening was higher than the 2 to 6% range reported in the major riociguat trials, including CHEST-1, MR BPA and RACE [4, 9, 10]. We attributed these disparities to the differences in baseline disease severity, as our cohort consisted exclusively of WHO functional class III patients with a mean intermediate-high REVEAL Lite 2 score of 7.3. Additionally, our patients demonstrated worse haemodynamics, with a mean baseline PVR of 11.1 Wood units and a cardiac output of 3.5 L/min, compared to 7.1–9.9 Wood units and 4–4.6 L/min in the riociguat groups of those landmark studies. This clinical profile indicates a population with more severe pulmonary hypertension and a greater predisposition to clinical worsening. Furthermore, a high proportion of our patients (69%) required long-term oxygen therapy, and three patients (19%) had interstitial lung disease. At baseline, these three patients had no clinical evidence of pulmonary hypertension. Each subsequently presented with acute pulmonary embolism, and further workup revealed new-onset pulmonary hypertension due to chronic thromboembolic pulmonary disease. Accordingly, they were managed as WHO Group 4 CTEPH. As patients with significant restrictive lung disease were excluded from the landmark trials, this affects the applicability of directly comparing our patient cohort to those trials. Nevertheless, such patients with advanced disease and multiple comorbidities, including significant pulmonary diseases, are often encountered in routine practice. Including these groups strengthened the real-world generalisability of our findings.

An important finding of this study was that despite significant improvements in haemodynamics and functional capacity, improvements in TAPSE and TRPG were not significant – a result consistent with a retrospective study of 11 Asian CTEPH patients, where riociguat significantly improved mPAP, PVR and WHO functional class but failed to demonstrate significant improvements in TRPG or tricuspid regurgitation grading [18]. It has been proposed that the elevated PVR in CTEPH leads to maladaptive remodelling of the right ventricle, and treatment strategies that reduce the afterload of the right ventricle are less effective in reversing the maladaptive remodelling of the right ventricle [19]. Our findings therefore suggest that while riociguat effectively reduced the afterload of the right ventricle as evidenced by PVR improvement, it may have limited capacity to reverse the established remodelling of the right ventricle.

It has been proposed that riociguat may complement BPA for CTEPH patients by targeting the microvasculopathy which cannot be addressed by BPA [9], formal recommendations regarding additional benefits of initiating riociguat either before or after BPA are currently lacking. The RACE trial ancillary 26-week follow-up study found that 6-month riociguat pretreatment reduced subsequent BPA complication rates by two-thirds (14% versus 42%) compared to upfront BPA, likely through hemodynamic optimisation before the procedure [10]. This finding suggested that riociguat pretreatment improved the safety profile of subsequent BPA in the management of CTEPH. On the other hand, evidence supporting the use of riociguat following BPA includes a randomised trial of 21 post-BPA CTEPH patients showed that riociguat significantly improved cardiac output and PVR at peak workload compared to controls [20]. The RACE trial ancillary study also showed that riociguat provided additional PVR reduction when added after suboptimal haemodynamic response to initial BPA [10]. Based on the evidence from landmark trials including CHEST-1, MR BPA and RACE [4, 9, 10], riociguat has been shown to improve clinical outcomes and haemodynamics in inoperable CTEPH. Despite this, off-label sildenafil remains widely used in many settings due to resource constraints. Our study contributes to the existing literature by demonstrating that switching from PDE5i to riociguat after the completion of BPA further improved both pulmonary haemodynamics and exercise capacity in patients with CTEPH.

### Limitations

This study has several important limitations that should be acknowledged. Firstly, the single-arm design without a control group introduced potential observer bias and could not account for placebo effects. Secondly, the single-centre design may limit the generalisability of the findings to broader populations. Thirdly, the small sample size limited the assessment of treatment response predictors and the detection of rare adverse events. Lastly, the selection of patients with residual pulmonary hypertension after BPA introduced a selection bias, as the study population primarily comprised BPA non-responders or incomplete responders. Consequently, this limited the generalisability of our findings to the broader post-BPA population. Overall, these limitations highlight the need for larger, multicentre and randomised controlled trials to validate the findings of this study.

## Conclusion

In patients with CTEPH, switching from PDE5i to riociguat improved pulmonary haemodynamics and functional capacity after BPA treatment. However, these benefits were accompanied by significant treatment-related adverse events, particularly hypotension, warranting the need for frequent home blood pressure monitoring and closer clinical surveillance. A careful risk-benefit assessment is essential when considering this switch, especially in patients with multiple comorbidities.

## Data Availability

The datasets analysed during the current study have been deposited in the UK data service that is accessible using the following link: https://doi.org/10.5255/UKDA-SN-857898.

## Abbreviations

6MWD: 6-min walking distance
BNP: Brain natriuretic peptide
BPA: Balloon pulmonary angioplasty
cGMP: Cyclic guanosine monophosphate
CI: Confidence interval
ERA: Endothelin receptor antagonist
mPAP: Mean pulmonary arterial pressure
NO: Nitric oxide
NT-proBNP: N-terminal prohormone of brain natriuretic peptide
PAH: Pulmonary arterial hypertension
PAWP: Pulmonary artery wedge pressure
PDE5i: Phosphodiesterase type 5 inhibitor
PEA: Pulmonary endarterectomy
PVR: Pulmonary vascular resistance
REVEAL: Registry to Evaluate Early and Long-Term PAH Disease Management
SD: Standard deviation
sGCs: Soluble guanylate cyclase stimulator
TAPSE: Tricuspid annular plane systolic excursion
TRPG: Tricuspid regurgitation pressure gradient
WHO: World Health Organisation
